# In-House IgM Dot-Blot Assay for Serodiagnosis of Human Leptospirosis: Development, Standardisation, and Performance Evaluation

**DOI:** 10.3390/microorganisms13061307

**Published:** 2025-06-04

**Authors:** Roberta Morozetti Blanco, Elaine dos Santos Lima, Juliana Maira Watanabe Pinhata, Angela Pires Brandao, Eliete Caló Romero

**Affiliations:** 1 Centro de Bacteriologia, Instituto Adolfo Lutz, São Paulo 01246-000, SP, Brazil; elaine.limabiom@yahoo.com.br (E.d.S.L.); pinhatajuliana@yahoo.com.br (J.M.W.P.); angela.brandao@ial.sp.gov.br (A.P.B.); eliete_romero@yahoo.com.br (E.C.R.); 2 Departamento de Doenças Infecciosas e Parasitárias, Faculdade de Medicina da Universidade de São Paulo, São Paulo 01246-903, SP, Brazil; 3 Instituto Oswaldo Cruz, Fundação Oswaldo Cruz (FIOCRUZ), Rio de Janeiro 21040-360, RJ, Brazil

**Keywords:** leptospirosis, serodiagnosis, whole-cell antigen, dot-blot

## Abstract

Laboratory confirmation of human leptospirosis relies on serological tests, with the microscopic agglutination test (MAT) as the reference. However, due to its complexity, there is a need for a simpler and more accessible diagnostic method. This study aimed to standardise and develop an IgM dot-blot test with a whole-cell antigen from saprophytic *Leptospira biflexa* serovar Patoc for diagnosing human leptospirosis. After checkerboard titration standardisation, IgM dot-blot was performed with paired serum samples from 124 MAT-confirmed leptospirosis cases and 143 serum samples from healthy and diseased individuals as the control group. Repeatability and reproducibility were also evaluated. An IgM dot-blot kit was then developed and compared to the Panbio^TM^ Leptospira IgM ELISA using 144 serum samples from patients with suspected leptospirosis. The IgM dot-blot showed a sensitivity of 58.1% and 96.0% when performed on acute and convalescent samples, respectively. Specificity was 94.4%. The repeatability and reproducibility of the IgM dot-blot showed 100% consistency. Comparison of IgM dot-blot and IgM ELISA showed almost perfect agreement, with a Kappa index of 0.81. The developed IgM dot-blot offers a robust alternative to existing methods, requiring minimal specialised equipment and fewer reagents than IgM ELISA. The good performance of this IgM dot-blot immunoassay makes it a promising tool for diagnosing human leptospirosis, potentially increasing diagnostic capacity, especially in places with limited resources.

## 1. Introduction

Leptospirosis is a globally distributed zoonosis with high incidence in tropical countries with humid climates. It disproportionately affects populations living in low-resource settings, where poor sanitation, unplanned urbanisation, and frequent flooding significantly increase the risk of exposure to contaminated water [1]. Vulnerable groups, particularly residents of informal settlements, often face barriers to timely diagnosis and medical care, which contributes to underreporting and worsens clinical outcomes [2,3].

In Brazil, the disease is endemic and remains a major public health concern, with a reported average case-fatality rate of 10.8% [4]. Notably, a significant increase in cases is usually observed during the rainy season, especially following episodes of heavy rainfall and flooding. In such contexts, the convergence of environmental and socioeconomic vulnerabilities creates ideal conditions for disease transmission [5,6].

Leptospirosis presents a wide spectrum of symptoms in humans, ranging from mild such as fever, headache, myalgia, anorexia, nausea, and vomiting, to severe forms like Weil’s syndrome, which is characterised by a triad of jaundice, renal failure, and haemorrhages—mainly pulmonary. Due to this broad range of clinical manifestations, leptospirosis is often misdiagnosed with other febrile illnesses such as dengue, yellow fever, influenza, and hepatitis,. Consequently, underreporting is common, highlighting the critical role of laboratory diagnosis for accurate case confirmation [7,8].

Currently, the microscopic agglutination test (MAT) is considered the reference test for laboratory diagnosis and provides the probable serogroup involved. However, this serological test is time-consuming and can only be used routinely by reference laboratories because it requires specialised technicians to maintain live cultures of several serovars of leptospires and carry out readings by dark field microscopy [7,9,10,11].

In light of these limitations, several screening tests have been proposed, showing a variety of sensitivity and specificity results [7,10,11,12,13,14,15,16,17]. This variability can be influenced by the characteristics of the study region, such as circulating strains and endemicity of the disease [18]. The whole-cell leptospiral antigen from the saprophytic *Leptospira biflexa* serovar Patoc is widely used in rapid tests because it is inexpensive and easy to use [15].

In Brazil, serological diagnosis is routinely performed by the Central Public Health Reference Laboratories (LACENs) using the commercial Panbio^TM^ Leptospira IgM ELISA kit (IgM ELISA) (Abbott Diagnostics, Yongin, South Korea), which is imported and distributed by the Ministry of Health and used as a screening test. This commercial IgM ELISA kit is also used in other countries for the diagnosis of human leptospirosis [19,20,21,22,23,24]. The MAT is used to confirm cases but is performed in only a few LACENs.

ELISA has the disadvantage of requiring specific equipment, such as microplate readers and plate washers. The acquisition, maintenance, and calibration of this equipment increase the cost of the test and make it difficult to perform in resource-limited settings [25]. For this reason, there is an urgent need for faster, simpler, and cheaper tests to diagnose human leptospirosis, which can be carried out in laboratories of any complexity and in regions with different socioeconomic levels.

Therefore, the purpose of this study was to standardise and develop a simple and easy-to-handle IgM dot-blot test with a whole-cell antigen from serovar Patoc for diagnosing human leptospirosis.

## 2. Materials and Methods

The present study adhered to the Standards for Reporting of Diagnostic Accuracy (STARD) [26,27], and the checklist is provided as Supplementary Table S1.

### 2.1. Study Design

**Figure 1:**
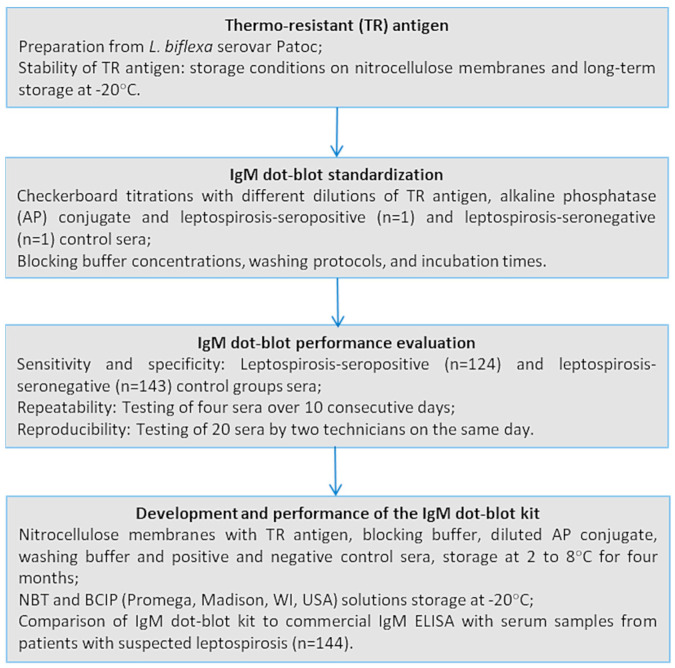
Flowchart of the study methodology.

This study aimed to standardise and evaluate the performance of an IgM dot-blot test, develop a kit, and check its stability over time. After standardisation through checkerboard titration, the IgM dot-blot was performed with leptospira-positive and leptospira-negative sera to determine its specificity and sensitivity, using MAT confirmation criteria. Inter- and intra-assay variability were also evaluated. This study was conducted retrospectively at the Instituto Adolfo Lutz (IAL), the LACEN of the State of Sao Paulo, Brazil, where human blood samples collected in health care units from the State are routinely tested by the commercial IgM ELISA and MAT for leptospirosis diagnosis. The flowchart showing the methodology of this study is available in Figure 1.

### 2.2. Routine Leptospirosis Diagnosis

#### 2.2.1. Panbio^TM^ Leptospira IgM ELISA (IgM ELISA)

IgM ELISA is conducted to screen serum samples for leptospirosis. If the result is reactive or inconclusive at any point during the disease, MAT is carried out to confirm the diagnosis. The assay is performed according to the manufacturer’s instructions. The cut-off calculation was determined by the mean absorbance of the cut-off calibrator multiplied by the calibration factor. The results are expressed as index values calculated by the ratio of sample absorbance to the cut-off value. A value > 1.1 index is considered reactive. Values of 0.9–1.1 and <0.9 index are considered inconclusive and non-reactive, respectively.

#### 2.2.2. Microscopic Agglutination Test (MAT)

MAT is conducted for disease confirmation following screening with IgM ELISA. Additionally, it is performed when paired sera are available or in death cases specimens. This test is carried out according to Goris and Hartskeerl [28], using the following serovars representing the main circulating serogroups [29,30]: Australis, Autumnalis, Bataviae, Canicola, Castellonis, Copenhageni, Cynopteri, Djasiman, Grippotyphosa, Hardjo, Hebdomadis, Icterohaemorrhagiae, Javanica, Panama, Patoc, Pomona, Pyrogenes, Sejroe, Tarassovi, and Wolfii. The strains were obtained from the National Leptospirosis Reference Centre, Fiocruz, Rio de Janeiro, RJ, and IAL—SP, Brazil. Titres ≥ 1:100 are considered reactive and laboratory-confirmed cases are defined according to established criteria for paired serology: seroconversion, i.e., the first sample negative and the second reactive with titres ≥ 1:200 or a four-fold titre difference between paired sera. The probable infecting serogroup can be determined if the higher serum titre is specific to a single serovar; otherwise, the infecting serogroup is considered inconclusive [31].

### 2.3. Serum Samples

The three groups of samples in this study were obtained from the specimens’ collection of IAL, where they are stored at −20 °C. All sera were tested as anonymous samples.

(i) Leptospirosis-seropositive group: Paired sera from 124 MAT-confirmed cases, collected at least five days apart, were tested to assess the IgM dot-blot performance. All the 124 acute-phase sera were negative by MAT. These sera were obtained from banked samples received by the Instituto Adolfo Lutz (IAL) between January 2007 and March 2018, which were submitted by healthcare facilities and hospitals across the state of São Paulo, Brazil, as part of routine clinical diagnostics. The probable infecting serogroups were Icterohaemorrhagiae (n = 72), Canicola and Cynopteri (n = 10 each), Australis and Sejroe (n = 4 each), Hebdomadis and Pyrogenes (n = 3 each), Bataviae (n = 2) and Grippotyphosa (n = 1). Fifteen sera showed inconclusive results with cross-reaction among the following serogroups: Australis, Autumnalis, Canicola, Cynopteri and Icterohaemorrhagiae (n = 10); Bataviae, Cynopteri and Hebdomadis (n = 2); Australis, Hebdomadis and Panama (n = 1); Autumnalis and Canicola (n = 1); Ballum and Grippotyphosa (n = 1).

The criteria used to select patients eligible for inclusion in this study were as follows:

Inclusion criteria: Patients from the state of São Paulo with leptospirosis confirmed by the Microscopic Agglutination Test (MAT) between 2007 and 2018.

Exclusion criteria: Cases with only a single serum sample available or cases in which both paired serum samples tested positive by MAT.

(ii) Leptospirosis-seronegative control group: this group, used to assess IgM dot-blot performance, consisted of 143 non-reactive sera by both IgM ELISA and MAT. Among these, 58 samples were collected from healthy persons at a blood donation bank, and 85 were collected from persons who tested positive for chikungunya (n = 1), dengue (n = 28), hantavirus disease (n = 3), hepatitis A (n = 4), Rocky Mountain spotted fever (n = 11), syphilis (n = 20), toxoplasmosis (n = 15), and yellow fever (n = 3).

(iii) Kit development group: 144 serum samples from patients suspected of having leptospirosis, received at IAL between March and July 2020 to be tested by IgM ELISA, were used to evaluate the performance of the IgM dot-blot kit.

### 2.4. IgM Dot-Blot Assay

#### 2.4.1. Antigen Preparation

Antigen was prepared from *Leptospira biflexa* serovar Patoc, strain Patoc I, belonging to the non-pathogenic serogroup Semaranga. This strain was obtained from the National Leptospirosis Reference Centre, Fiocruz, RJ.

Thermo-resistant antigen (TR) was prepared according to Faine [32], with few modifications. A Patoc I culture was maintained at 30 °C for 5–7 days in 5.5 mL of Ellinghausen-McCullough-Johnson-Harris (EMJH) liquid medium (Difco, Bergen, NJ, USA), centrifuged at 10,000× *g* at 4 °C for 30 min and washed twice with PBS pH 7.4. The sediment was suspended in PBS pH 7.4 and kept at 4 °C overnight under gentle shaking. The suspension was boiled for 30 min. After centrifugation at 10,000× *g* at 4 °C for 30 min, the sediment was resuspended in PBS pH 7.4 and adjusted to McFarland standard 10. The protein concentration was determined by spectrophotometry with the Bradford method [33], using Bio-Rad Protein Assay kit II (Bio-Rad, Hercules, CA, USA). The TR antigen was kept at 4 °C for 7 days and then stored at −20 °C.

#### 2.4.2. Assay Protocol

The IgM dot-blot test was carried out according to Blanco et al. [34] with modifications. For the test, 1 µL of TR antigen was dotted onto 0.45 µm nitrocellulose membrane strips of 0.5 cm × 0.5 cm (Bio-Rad, Hercules, CA, USA). After air drying, the membranes were blocked with skimmed milk (Scharlab, Barcelona, Spain) for 30 min at room temperature. The membranes were then incubated with the serum samples diluted in blocking buffer under continuous shaking at room temperature and washed with 600 µL of wash buffer. Next, the membranes were incubated with alkaline phosphatase (AP)-conjugated goat anti-human IgM (Sigma, St. Louis, MO, USA) at room temperature under continuous shaking. The membranes were rewashed. The colour was developed with nitroblue tetrazolium (NBT) and 5-bromo-4-chloro-3-indolyl phosphate (BCIP) as substrate (Promega, Madison, WI, USA) for up to 15 min. The reaction was stopped by washing the membrane with distilled water for 1 min. The development of a purple dot on the nitrocellulose membrane was considered reactive. The non-appearance of coloured dots was considered non-reactive. The test was inspected visually and interpreted by two independent observers blind to all sera information.

#### 2.4.3. Standardisation

Two serum samples, one from a MAT-confirmed leptospirosis case and the other from a healthy blood donor of the control group, were used to assess the standardisation of the IgM dot-blot test.

Checkerboard titrations were carried out with the TR antigen in five two-fold dilutions from 1:2 to 1:32 in Phosphate-Buffered Saline (PBS) pH 7.2, and six sera dilutions (1:10, 1:50, 1:100, 1:200, 1:400, and 1:800). The conjugate was tested at 1:1000, 1:2000, and 1:3000 dilutions.

The skimmed milk was tested at 1%, 2%, 3%, 4%, and 5% in PBS pH 7.2 (M-PBS) and in PBS pH 7.2 containing 0.1% tween 20 (PBS-T), named M-PBS-T. These M-PBS and M-PBS-T concentrations were used to block the membranes, and dilute the sera and conjugate.

The membrane washing steps were evaluated using PBS pH 7.2 and PBS-T, with the protocol repeated three, four, and five times.

The incubation times of the membranes with the serum samples and the conjugate were evaluated by leaving the membranes incubated for 30 and 60 min.

#### 2.4.4. Performance

To estimate the sensitivity and specificity of the test after standardisation, the IgM dot-blot protocol was carried out using 124 paired sera from individuals with confirmed leptospirosis, as well as sera from the control group, comprising 58 healthy persons and 85 individuals with other diseases presenting similar symptoms to leptospirosis.

To check repeatability, four sera from persons with suspected leptospirosis were selected based on IgM ELISA results: two reactive and two non-reactive samples (close to and far from the cut-off). The IgM dot-blot test was carried out in duplicate over 10 consecutive days, excluding weekends. If an unexpected result occurred, all results from that run were rejected. No more than one out of 10 runs could be rejected.

To calculate reproducibility, 10 reactive and 10 non-reactive sera (results close to or far from the cut-off) were selected as in the repeatability evaluation. Two technicians carried out the IgM dot-blot test on these samples on the same day.

#### 2.4.5. Stability of the TR Antigen

To verify the stability of the dotted antigen, unblocked and blocked membranes with 3% L-PBS-T were stored at room temperature, 4 °C and −20 °C. These were then tested with positive and negative control sera at 3, 6, 9, and 12 months after spotting the antigen.

Additionally, the stability of the TR antigen stored at −20 °C was evaluated using the same positive control serum annually for up to five years.

### 2.5. Development and Performance of the IgM Dot-Blot Kit

An IgM dot-blot kit was prepared to contain nitrocellulose membranes dotted with TR antigen, blocking buffer, diluted AP conjugate, washing buffer, NBT and BCIP solutions, substrate buffer, and positive and negative control sera. This kit was kept refrigerated, except for the NBT and BCIP solutions, which were kept at −20 °C for 4 months. After this period, 144 serum samples from patients with suspected leptospirosis received at IAL between March and July 2020 were tested in parallel by IgM ELISA and IgM dot-blot. All samples were analysed by the same analyst, who was blind to all the information.

### 2.6. Statistical Analysis

The sensitivity of the IgM dot-blot was calculated separately for the acute and convalescent phases of MAT-confirmed leptospirosis. Specificity was estimated using MAT-negative sera from healthy individuals or those with other diseases (control group). The percentage of agreement was calculated to assess repeatability and reproducibility. Kappa statistics were used to evaluate the agreement of visual readings by two independent observers, the reproducibility of the test and the comparison of the index test to the IgM ELISA. Statistical analyses were performed using MedCalc for Windows, version 15.0 (MedCalc Software, Ostend, Belgium). Kappa was calculated using the GraphPad QuickCalcs website, and the results were interpreted as follows: values ≤ 0 no agreement, 0.01–0.20 slight, 0.21–0.40 fair, 0.41–0.60 moderate, 0.61–0.80 substantial, and 0.81–1.00 almost perfect agreement [35,36].

## 3. Results

**Figure 2:**
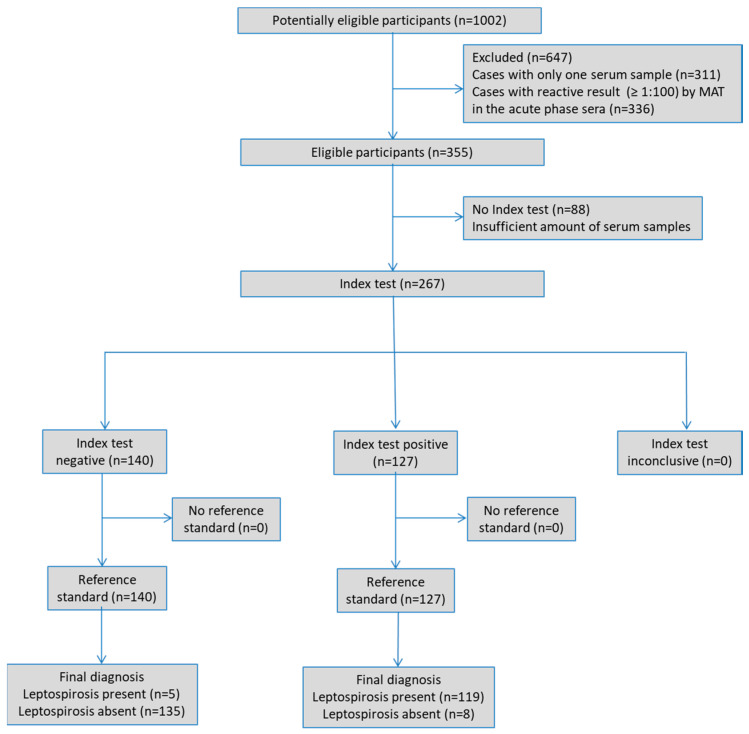
Standards for Reporting of Diagnostic Accuracy (STARD) flow chart of the 124 MAT-confirmed cases from Leptospirosis-seropositive group and 143 cases from Leptospirosis-seronegative control group.

The median age of confirmed cases of leptospirosis was 36.6 years (range 9–74), and 109 (87.9%) were male. Since the index test was performed on banked samples, the clinical characteristics of the participants were not available. STARD diagram illustrating the flow of participants through the study is presented in Figure 2.

### 3.1. Antigen Yield and Dot-Blot Standardisation

**Figure 3:**
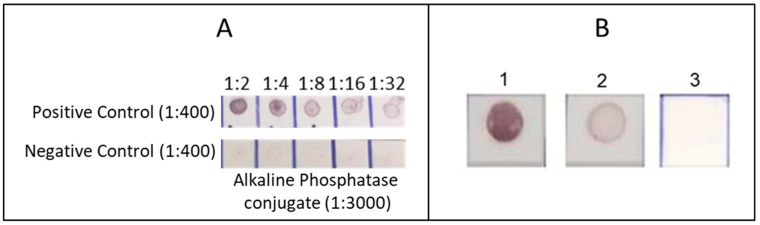
Representative results of IgM dot-blot. Column (**A**): Checkerboard titrations of the TR antigen with positive and negative control sera and alkaline phosphatase conjugate. Column (**B**): Representative reactions showing: 1–2 reactive results; 3 non-reactive results.

A 5.5 mL culture of Patoc I strain in EMJH medium yielded 0.5 mL of TR antigen, with a protein concentration of 130 μg/mL. The optimal conditions for the IgM dot-blot assay were obtained with the TR antigen diluted 1:4, corresponding to 32.5 ng of protein (with 1 μL applied per dot); serum diluted 1:400; AP conjugate diluted 1:3000; four repetitions for each washing step with PBS-T; 3% skimmed milk in PBS-T (3% L-PBS-T) as the blocking buffer for membranes and the diluent for sera and conjugate; and an incubation time of 30 min for both serum and conjugate. Representative results of checkerboard titration of the TR antigen and the IgM dot-blot results after standardisation are shown in Figure 3.

### 3.2. Performance

The IgM dot-blot reacted with antibodies against all probable infecting serogroups as determined by MAT. Serum samples from 124 acute-phase leptospirosis cases that were MAT non-reactive exhibited 58.1% reactivity in the IgM dot-blot. This sensitivity increased to 96.0% when the assay was performed on convalescent-phase samples (Table 1). The specificity of the test was 94.4%.

For 5 out of 124 persons (4.0%), both acute and convalescent sera were non-reactive by the IgM dot-blot. Four of these had samples collected 25 days or more after the onset of symptoms, with MAT titres ranging from 1:200 to 1:800. The remaining cases had samples collected within 14 days of symptoms onset and showed seroconversion of 1:200. 

When compared to IgM ELISA (n = 62/124; 50.0%) conducted on acute-phase sera, the IgM dot-blot sensitivity was slightly higher (n = 72/124); 58.1%.

The agreement of the visual readings by two independent observers was almost perfect, presenting a 0.97 (95% CI 0.95–0.99) kappa score (n = 386/391; 98.7%).

All the results of the tests carried out to check the repeatability (10 replicates with four serum samples in duplicate by the same technician) and reproducibility (twenty serum samples tested by two technicians) were both 100%. In addition, the agreement between the tests carried out by two technicians showed a Kappa index of 1.00.

### 3.3. Stability

The membranes remained stable for up to 12 months in all storage conditions. Furthermore, the TR antigen stored at −20 °C consistently showed reactive results with the positive control serum across five years of annual IgM dot-blot tests demonstrating good stability.

### 3.4. Development and Performance of the IgM Dot-Blot Kit

The IgM dot-blot kit, prepared four months prior to testing, contained membrane strips dotted with 1 µL of TR antigen diluted 1:4 in PBS pH 7.2, 3% L-PBS-T, AP conjugate diluted 1:3000 in 3% L-PBS-T, PBS-T solution for washing, 5% NBT, 5% BCIP, substrate buffer, and positive and negative control sera. Analysis of the 144 blinded serum samples from patients with suspected leptospirosis, tested using both the IgM ELISA and the IgM dot-blot kit showed almost perfect agreement (Kappa = 0.81) with 84.6% sensitivity, 96.6% specificity, and 94.4% accuracy (Table 2).

Among four serum samples reactive in the IgM ELISA but non-reactive in the IgM dot-blot, one case was identified as missed by the IgM dot-blot test. This was confirmed when seroconversion was detected via the MAT in a subsequent sample from the same patient. The other three samples were MAT non-reactive. On the other hand, at least one of the four serum samples non-reactive in the IgM ELISA but reactive in the IgM dot-blot was missed by IgM ELISA. This sample, which was from a deceased patient, showed an antibody titre of 1:200 in MAT. MAT was not performed on the other three samples.

## 4. Discussion

This study evaluated an IgM dot-blot test using a thermo-resistant antigen from the saprophytic *Leptospira* serovar Patoc and AP as a standardised conjugate.

Serological tests are the most widely used methods for leptospirosis diagnosis and are essential for the correct treatment of patients and for epidemiological surveillance and prevention of the disease. Serovar Patoc is commonly used in serological tests for leptospirosis because it is safer and easier to culture than pathogenic serovars. Additionally, its ability to cross-react with antibodies against multiple pathogenic *Leptospira* serovars makes it a valuable broad-spectrum screening antigen. Dot-blot tests use a small quantity of reagents and do not require specialised equipment, making them an advantage over ELISA tests.

The IgM dot-blot test developed in this study was fast, with a turnaround time of approximately 1 h 45 min, comparable to that of IgM ELISA. It was also simple to perform, requiring a few consumables and only a shaker. The incubations were carried out at room temperature, and the results were read visually. Although visual results are subjective, the test was reliable, with a kappa index of 0.97 between two observers. In addition, it used whole-cell antigens from only one saprophytic serovar, which presents less biological risk in its handling. The TR antigen was easy to prepare, requiring basic laboratory equipment, like micropipettes, water baths, and centrifuges. The antigen yield was sufficient for approximately 2000 reactions, which avoided additional antigen preparations.

The sensitivity showed by the IgM dot-blot test was 58.1% in the acute phase of the disease in samples with non-reactive MAT results. This sensitivity was slightly higher than that shown by the commercial IgM ELISA (50.0%) with the same serum samples. This lower sensitivity in the acute phase is expected, as antibodies typically become detectable five to seven days after the onset of symptoms [7]. However, it is crucial for screening tests for the diagnosis of leptospirosis to have higher sensitivity than MAT in the early stages of the disease to allow for prompt treatment, thereby reducing hospitalisation and mortality rates, with MAT used for subsequent confirmation. The sensitivity in convalescent sera was 96.0% and specificity was 94.4% considering the entire control group. These results are comparable to those of other genus-specific tests for diagnosing human leptospirosis, including Panbio^TM^ Leptospira IgM ELISA (Abbott Diagnostics, Yongin, South Korea). Previous studies have reported sensitivity rates ranging from 17.7% to 100% and specificity rates ranging from 39.9% to 100% [15,21,23,37,38,39,40].

The IgM dot-blot showed reactive results with all the different serogroups classified by MAT, suggesting that it is genus-specific and, therefore, suitable as a screening test for the diagnosis of human leptospirosis caused by the main serogroups circulating in Brazil.

The evaluation of repeatability and reproducibility of the test to assess the consistency of results when the test is repeated [41,42] showed that the IgM dot-blot test is highly consistent, with 100% repeatability and reproducibility.

The stability tests with dotted membranes revealed that the IgM dot-blot assay remained functional for at least 12 months under all storage conditions, including room temperature. Notably, the immunoreactivity of the TR antigen stored at −20 °C remained fully functional for at least five years. This remarkable property is advantageous for manufacturing purposes, facilitating the use of this immunoassay, especially in remote areas. If implemented, the TR antigen could be prepared in a well-equipped laboratory such as a LACEN facility. An IgM dot-blot kit containing ready-to-use dotted membrane strips could then be shipped to remote laboratories with minimal infrastructure under very basic cold-chain conditions.

Using the kit prepared in this study, only a small number of samples (n = 8/144) presented discordant results between IgM dot-blot and IgM ELISA. The high concordance (94.4%) of these two screening tests, together with the almost perfect agreement score (Kappa = 0.81), shows the non-inferiority of this new test compared to the IgM ELISA, making it capable of detecting human leptospirosis as effectively as the screening test currently used in Brazil. If the results presented herein are confirmed, mainly by prospective studies with a larger number of samples at different laboratories, this new test could be implemented as a serological screening test for routine diagnosis of human leptospirosis in Brazil. This would impact the cost of the leptospirosis diagnosis, especially since the IgM ELISA kit currently used in Brazil is imported.

This study has some limitations that should be acknowledged. First, it was not possible to obtain the clinical data of the patients. The samples received in the laboratory for diagnosing human leptospirosis were from patients with moderate to severe symptoms, who are often hospitalised. These samples may not adequately represent the general population of leptospirosis patients, especially those with mild or asymptomatic forms of the disease. Consequently, the results may not be generalisable to all cases of leptospirosis, and the sensitivity and specificity rates of the diagnostic test evaluated may differ in patients with less severe clinical manifestations. However, it is important to note that patients with the most severe symptoms are the ones in greater need of early and rapid diagnosis due to the urgency of treatment and the higher likelihood of death.

Additionally, all clinical samples used in this study were obtained from patients who received medical care in the state of São Paulo, Brazil. Although this region is characterised by high endemicity and diverse epidemiological scenarios, and the assay employs the Patoc serovar as an antigen—widely known for its ability to elicit cross-reactions with antibodies against multiple pathogenic serovars—this geographic concentration may limit the generalisability of the findings. Further studies involving samples from other geographic areas are necessary to validate the assay’s performance across a broader range of epidemiological contexts.

The IgM dot-blot test developed in this study for screening human leptospirosis meets the requirements for implementation in laboratories performing less complex testing. It is simple and reasonably quick to perform, does not require costly equipment or consumables, and the antigen is easy to prepare with good yield and remarkable stability. Additionally, it is more affordable than the IgM ELISA, which could potentially be replaced if further studies confirm their high concordance.

## Figures and Tables

**Table 1:** Performance of the IgM dot-blot for diagnosing human leptospirosis using MAT as the comparison standard for leptospirosis confirmed cases.

IgM Dot-Blot Performance	MAT-Confirmed Cases and Controls		
	No. Reactive/Total	Rates	(95% CI)
Sensitivity			
Acute phase	72/124	58.1%	(48.9–66.9)
Convalescent phase	119/124	96.0%	(90.8–98.7)
Specificity			
Healthy individuals	6/58	89.7%	(78.8–96.1)
Other diseases	2 */85	97.6%	(91.8–99.7)
Total control group	8/143	94.4%	(89.3–97.6)

MAT, microscopic agglutination test; 95%CI, 95% confidence interval; * 2 patients with toxoplasmosis.

**Table 2:** Performance of IgM dot-blot in serum samples from patients suspected of leptospirosis using Panbio^TM^ Leptospira IgM ELISA as the comparison standard.

Performance Measures	No. Matching/Total	Estimate	95% Confidence Interval
Sensitivity	22/26	84.6%	65.1–95.6%
Specificity	114/118	96.6%	91.6–99.1%
Accuracy	136/144	94.4%	89.4–97.6%
Agreement Kappa index	136/144	0.81	0.69–0.94

## Data Availability

The original contributions presented in this study are included in the article/Supplementary Materials. Further inquiries can be directed to the corresponding author.

## Supplementary Materials

The following supporting information can be downloaded at https://www.mdpi.com/article/10.3390/microorganisms13061307/s1, Table S1: Standards for reporting of diagnostic accuracy (STARD) checklist.

## Abbreviations

The following abbreviations are used in this manuscript:

MAT	Microscopic Agglutination Test
LACENs	Central Public Health Reference Laboratories
STARD	Standards for Reporting of Diagnostic Accuracy
TR	Thermo-resistant antigen
EMJH	Ellinghausen-McCullough-Johnson-Harris
AP	Alkaline Phosphatase
NBT	Nitroblue tetrazolium
BCIP	5-bromo-4-chloro-3-indolyl phosphate
PBS	Phosphate-Buffered Saline
PBS-T	PBS + 0.1% tween 20
M-PBS	Skimmed milk in PBS
M-PBS-T	Skimmed milk in PBS-T
