# Supplementary material for: In-House IgM Dot-Blot Assay for Serodiagnosis of Human Leptospirosis: Development, Standardisation, and Performance Evaluation
Source: Microorganisms. 2025 Jun 4;13(6):1307. doi: 10.3390/microorganisms13061307 (PMC12195038; doi:10.3390/microorganisms13061307)
Supplement: Supplementary file 1 [file microorganisms-13-01307-s001.zip › microorganisms-3648360-supplementary.pdf]

Table S1. Standards for reporting of diagnostic accuracy (STARD) checklist.

Section and Topic	No	Item	Reported on page
**TITLE OR ABSTRACT**			
	**1**	Identification as a study of diagnostic accuracy using at least one measure of accuracy (such as sensitivity, specificity, predictive values, or AUC)	Page 2
**ABSTRACT**			
	**2**	Structured summary of study design, methods, results, and conclusions (for specific guidance, see STARD for Abstracts)	Page 2
**INTRODUCTION**			
	**3**	Scientific and clinical background, including the intended use and clinical role of the index test	Pages 3-4
	**4**	Study objectives and hypotheses	Page 4
**METHODS**			
Study design	**5**	Whether data collection was planned before the index test and reference standard were performed (prospective study) or after (retrospective study)	Page 4
Participants	**6**	Eligibility criteria	Pages 6-7
	**7**	On what basis potentially eligible participants were identified (such as symptoms, results from previous tests, inclusion in registry)	Pages 6-7
	**8**	Where and when potentially eligible participants were identified (setting, location and dates)	Pages 6-7
	**9**	Whether participants formed a consecutive, random or convenience series	Not Applicable
Test methods	**10a**	Index test, in sufficient detail to allow replication	Pages 7-8

Section and Topic	No	Item	Reported on page
	**10b**	Reference standard, in sufficient detail to allow replication	Page 6
	**11**	Rationale for choosing the reference standard (if alternatives exist)	Page 6
	**12a**	Definition of and rationale for test positivity cut-offs or result categories of the index test, distinguishing pre-specified from exploratory	Page 8
	**12b**	Definition of and rationale for test positivity cut-offs or result categories of the reference standard, distinguishing pre-specified from exploratory	Page 6
	**13a**	Whether clinical information and reference standard results were available to the performers/readers of the index test	Page 8
	**13b**	Whether clinical information and index test results were available to the assessors of the reference standard	Page 8
Analysis	**14**	Methods for estimating or comparing measures of diagnostic accuracy	Pages 9-10
	**15**	How indeterminate index test or reference standard results were handled	Not Applicable
	**16**	How missing data on the index test and reference standard were handled	Not Applicable
	**17**	Any analyses of variability in diagnostic accuracy, distinguishing pre-specified from exploratory	Pages 9-10
	**18**	Intended sample size and how it was determined	Not Applicable
**RESULTS**			
Participants	**19**	Flow of participants, using a diagram	Figure 2

Section and Topic	No	Item	Reported on page
	**20**	Baseline demographic and clinical characteristics of participants	Page 10
	**21a**	Distribution of severity of disease in those with the target condition	Information not available
	**21b**	Distribution of alternative diagnoses in those without the target condition	Information not available
	**22**	Time interval and any clinical interventions between index test and reference standard	Not Applicable
Test results	**23**	Cross tabulation of the index test results (or their distribution) by the results of the reference standard	Table 1
	**24**	Estimates of diagnostic accuracy and their precision (such as 95% confidence intervals)	Pages 12-13; Table 1
	**25**	Any adverse events from performing the index test or the reference standard	Not Applicable
**DISCUSSION**			
	**26**	Study limitations, including sources of potential bias, statistical uncertainty, and generalizability	Page 16
	**27**	Implications for practice, including the intended use and clinical role of the index test	Pages 16-17
**OTHER INFORMATION**			
	**28**	Registration number and name of registry	Not Applicable
	**29**	Where the full study protocol can be accessed	Not Applicable
